# Genome-wide associations and epistatic interactions for internode number, plant height, seed weight and seed yield in soybean

**DOI:** 10.1186/s12864-019-5907-7

**Published:** 2019-06-26

**Authors:** Teshale Assefa, Paul I. Otyama, Anne V. Brown, Scott R. Kalberer, Roshan S. Kulkarni, Steven B. Cannon

**Affiliations:** 1 ORISE Fellow, USDA-ARS, Corn Insects and Crop Genetics Research Unit, Ames, Iowa USA; 2 Agronomy Department, Iowa State University, Ames, IA USA; 3 USDA-ARS, Corn Insects and Crop Genetics Research Unit, Ames, Iowa USA

**Keywords:** GWAS, GWES, Epistasis, Internode number, Plant height, Seed weight, Seed yield per plant, QTL, Soybean

## Abstract

**Background:**

Breeding programs benefit from information about marker-trait associations for many traits, whether the goal is to place those traits under active selection or to maintain them through background selection. Association studies are also important for identifying accessions bearing potentially useful alleles by characterizing marker-trait associations and allelic states across germplasm collections. This study reports the results of a genome-wide association study and evaluation of epistatic interactions for four agronomic and seed-related traits in soybean.

**Results:**

Using 419 diverse soybean accessions, together with genotyping data from the SoySNP50K Illumina Infinium BeadChip, we identified marker-trait associations for internode number (IN), plant height (PH), seed weight (SW), and seed yield per plant (SYP). We conducted a genome-wide epistatic study (GWES), identifying candidate genes that show evidence of SNP-SNP interactions. Although these candidate genes will require further experimental validation, several appear to be involved in developmental processes related to the respective traits. For IN and PH, these include the Dt1 determinacy locus (a soybean meristematic transcription factor), as well as a pectinesterase gene and a squamosa promoter binding gene that in other plants are involved in cell elongation and the vegetative-to-reproductive transition, respectively. For SW, candidate genes include an ortholog of the AP2 gene, which in other species is involved in maintaining seed size, embryo size, seed weight and seed yield. Another SW candidate gene is a histidine phosphotransfer protein - orthologs of which are involved in cytokinin-mediated seed weight regulating pathways. The SYP association loci overlap with regions reported in previous QTL studies to be involved in seed yield.

**Conclusions:**

This study further confirms the utility of GWAS and GWES approaches for identifying marker-trait associations and interactions within a diverse germplasm collection.

**Electronic supplementary material:**

The online version of this article (10.1186/s12864-019-5907-7) contains supplementary material, which is available to authorized users.

## Background

Soybean (*Glycine max* [L] Merr.) is the world’s largest single source of vegetable protein and oil, accounting for more than 50% of world edible oil (Soystats 2013, http://www.soystats.com). Soybean is also a valuable component of many agricultural systems due to its N-fixing capacity. Improvement of seed yield is a major objective in soybean breeding. Seed yield (seed yield per plant, SYP) is a complex trait and is influenced by many developmental traits including seed weight (SW), internode number (IN) and plant height (PH). Like seed yield, these developmental traits are also quantitatively inherited. For example, SW is influenced by numerous physiological and morphological components [1]. Internode number and plant height affect seed yield via their impact on important traits including lodging and adaptability in soybean [2].

Many linkage mapping studies in soybean have been curated and compiled at SoyBase (https://www.soybase.org), collectively resulting in approximately 250, 200 and 30 QTLs for SW, PH and IN, respectively ([3] https://www.soybase.org). Significant, positive correlations have also been reported between PH and IN [3] as well as SW and SYP [4, 5]. Recent mapping studies have identified associations among QTLs related to seed yield and seed weight [2, 6, 7]. However, in general, QTL studies for yield and seed weight have not resulted in the detection of candidate genes, due to the typically low genetic resolution of biparental QTL studies [6].

Plant height and internode number have significant correlations with flowering and maturity traits, which are important agronomic traits associated with adaptability and productivity in soybean [8]. Chang et al. [3] identified 34 loci for PH and 30 loci for node number via genome wide association studies (GWAS) in 368 soybean accessions. This study also confirmed that IN and PH are correlated (*r* = 0.49). Similarly, Fang et al. [9] phenotyped 809 accessions for eighty-four agronomic traits and identified 245 significant loci, of which 95 genetically interacted with other loci. They also reported correlation of IN and PH (r = 0.6). IN and PH are polygenic traits controlled by many loci of small effect [3, 8]. The molecular mechanisms of some genes involved in IN and PH have been reported – for example, *Dt1* is a meristematic transcription factor, orthologous to the *A. thaliana Tfl1* gene [10], and *E2* is an ortholog of GIGANTEA, which functions upstream of CONSTANS (CO) and FLOWERING LOCUS T (FT) in *A. thaliana* [11]. A linkage mapping study by Sun et al. [12] showed various QTL for plant height at different growth stages. Similarly, Chang et al. [3] reported that several loci of IN and PH were captured at different growth stages in soybean. Several other studies that associated developmental quantitative traits with genetic markers have been reported in soybean [3, 13, 14].

GWAS methods provide a powerful approach for discovering candidate genes associated with complex traits [3, 15–17]. They have identified QTLs in many crop species, including rice, maize, and soybean. GWAS complements QTL studies by offering a way to identify more association regions with greater precision – albeit depending on the number, diversity and genetic structure of the germplasm accessions.

GWAS mainly addresses additive genetic effects; however, these only explain a portion of the heritability estimates for complex traits. Recent studies have revealed that both additive and epistatic interactions have measurable effects on the genetic architecture of soybean diseases such as sclerotinia stem rot, and sudden death syndrome [18, 19]. The combination of additive genetic and epistatic effects was able to explain additional phenotypic variations. We have used a “genome wide epistatic study” (GWES) approach to complement the more widely-used GWAS analysis and provide a fuller understanding of the genetic architecture of complex traits. In particular, GWES helps reveal the genetic basis of IN, PH, SW and SYP in soybean.

## Results

### Measurements from field evaluation

Significant differences (*P* < 0.05) were found among the 419 genotypes for all four traits (IN, PH, SW, SYP), measured at three locations. The IN (measured on the main stem) ranged from 8.3 to 17.8 with an average of 13.8. The PH ranged from 22.6 to 144.8 cm with an average of 70.3 cm. The SW ranged from 2.9 to 29.3 g with an average of 12.6 g. The SYP ranged from 7.6 to 81.0 g with an average of 33.9 g (Table 1). The population had approximately normal distribution for all four traits (Additional file 1: Figure S1). The heritability estimate was found to be moderate to high for each trait (65–75%), suggesting that genetic effects played a significant role in each trait’s expression (Table 1).

**Table 1:** Phenotypic variation of internode number, plant height, 100 seed weight, and seed yield per plant, based on genotype performance across locations

Trait	Mean ± SEM	Range	Heritability (%)
Internode number (count)	13.8 ± 0.36	8.3–17.8	65
Plant height (cm)	70.3 ± 8.6	22.6–144.8	70
100 seed weight (gm)	12.6 ± 0.64	2.9–29.3	75
Seed yield per plant (gm)	33.9 ± 5.0	7.6–81	67

SEM = Standard Error of the Mean

### Linkage disequilibrium (LD)

The linkage disequilibrium (LD) decay rate for euchromatin and heterochromatin was 238 kb and 1648 kb, respectively (Additional file 2: Figure S2). The average marker density varied over different chromosomes, from 39 kb per SNP in chromosome 1 (Gm01) to 19 kb per SNP in chromosome 14 (Gm14). From the total SNPs used for the soybean genome, 77% were found in euchromatic regions where 78% of the predicted genes occurred in the chromosome ends; this is the region which accounts for most genetic recombination events.

### GWAS and candidate gene prediction

**Fig. 1:**
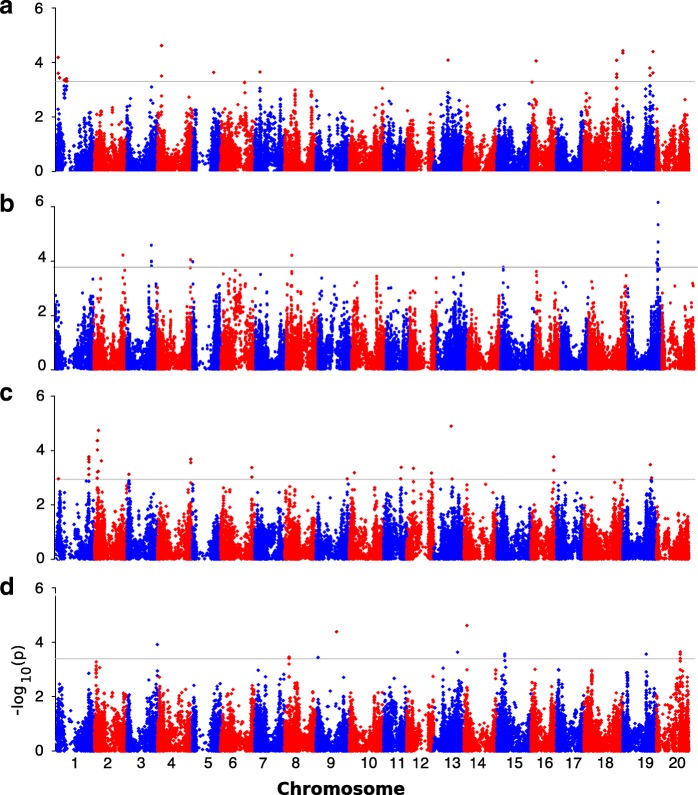
Manhattan plots of GWAS for internode number. The horizontal black line in each panel shows a selected genome-wide significance threshold at FDR < 0.05. The panels give GWAS values for (**a**) internode number (IN), (**b**) plant height (PH), (**c**) seed weight (SW), and (**d**) seed yield per plant (SYP). See Table 2 for regions and candidate genes

The best linear unbiased prediction (BLUP) of each PI line’s performance across three environments was used in a mixed linear model (MLM). This strategy was used to help minimize the rate of false positives [20]. A total of 75 significant SNPs (*P* < 0.05) were identified for the IN, PH, SW, and SYP traits (Fig. 1 a, b, c and d). The trait variation explained by each marker (R^2^) varied from 0.03 to 0.11.

For all loci showing significant GWA, we examined annotations of genes both within regions defined by nonsignificant SNPs that flanked significant GWA SNP peaks, and within regions defined by 100 kb windows around significant GWA SNPs. In addition, we compared these results with QTL intervals for related traits reported for these regions. The count of genes falling within these two types of regions around each locus is given in Table 2, and details about all genes and loci in Additional files 3 and 4. The average number of genes found within regions around one or more significant SNPs and between non-significant SNPs is 5.1 genes (max 33, min 0), and the average number of genes found in regions defined by 100 kb windows around significant GWA SNPs is 22.3 (max 37, min 3). SNPs showing stable interactions across all three loci are indicated in Table 2 and Additional file 3.

**Table 2:** Summary of GWAS regions, genes of interest, and overlapping QTLs. Counts of candidate genes are given for regions defined by two methods: by flanking non-significant markers (column “Genes in region - A”) and within a window defined by the significant SNP(s) plus 100 kb before and after the significant SNP(s) (column “Genes in region - B”). Known QTLs are abbreviated IN: Node number; PH: Plant height; SY: seed yield; SW: seed weight. Also see a more detailed version of the table in Additional file 3 (including full *QTL names), and a list of all genes within GWAS regions in Additional file 4

Start SNP	End SNP	Chrom	Gene count A	Gene count B	P-value	stable 3 locs	Genes of special interest	Annotation	Known QTLs*
Internode Number (IN)									
ss715578706	ss715578717	Gm01	8	24	7E-05		Glyma.01 g022500	AP2-like ethylene-responsive TF	NN
ss715579430	ss715579430	Gm01	1	17					
ss715578432	ss715578590	Gm01	3	3					
ss715578480	ss715578480	Gm01	4	9					
ss715578432	ss715578590	Gm01	4	3	0.0004		Glyma.01 g074000	ubiquitin protein ligase	NN, PH
ss715589109	ss715589108	Gm04	0	25					
ss715590708	ss715590708	Gm05	6	24					
ss715598728	ss715598728	Gm07	3	21					
ss715614000	ss715614000	Gm13	3	23	8E-05		Glyma.13 g101400	Putative lysophospholipase	NN, PH
ss715625429	ss715625429	Gm16	0	21					
ss715623458	ss715623458	Gm16	3	22	0.0005		Glyma.16 g016400	NAC domain containing protein 62	NN, PH
ss715631395	ss715631424	Gm18	14	24	8E-05	X	Glyma.18 g203800	Metallopeptidase protein	PH
ss715635223	ss715635190	Gm19	4	29	4E-05		Glyma.19 g002900	NAC domain-containing protein 8-like	PH
ss715635454	ss715635456	Gm19	5	24	4E-05	X	Glyma.19 g194300	Phosphatidylethanolamine binding prot (Dt1)	PH
ss715635024	ss715635024	Gm19	1	22	0.0002		Glyma.19 g146000	squamosa promoter binding	PH
same	same						Glyma.19 g145700	pectinesterase	PH
same	same						Glyma.19 g145400	plant phosphoribosyltransferase protein	PH
Plant Height (PH)									
ss715582992	ss715582994	Gm02	3	23	9E-05	X	Glyma.02G245600	Gibberellin-regulated family protein	PH
ss715585780	ss715585783	Gm03	10	25	4E-05				
ss715585781	ss715585784	Gm03	9	24	0.0002				
ss715585788	ss715585790	Gm03	8	23	0.0002				
ss715588701	ss715588703	Gm04	3	25	0.0001				
ss715592575	ss715592573	Gm05	1	20	0.0002				
ss715602866	ss715602868	Gm08	6	36	9E-05				
ss715635355	ss715635357	Gm19	3	26	0.0001	X	Glyma.19G187800	pectinesterase 11	PH, SL
ss715635405	ss715635407	Gm19	3	17	7E-05	X	Glyma.19G192400	ethylene-responsive transcription factor 12	PH, SL
same	same					X	Glyma.19G192700	growth-regulating factor 4	PH, SL
ss715635424	ss715635459	Gm19	17	36	1E-06	X	Glyma.19 g194300	Phosphatidylethanolamine binding prot (Dt1)	PH, SL
Seed Weight (SW)									
ss715579653	ss715579702	Gm01	18	26					
ss715583620	ss715583627	Gm02	5	35					
ss715582341	ss715582351	Gm02	5	26	8E-06		Glyma.02 g04660	histidine kinase 1	SW
ss715584321	ss715584321	Gm02	1	16					
ss715588755	ss715588756	Gm04	3	27	5E-05	X	Glyma.04 g228200	Late embryogenesis protein (LEA)	SW
ss715594571	ss715594571	Gm06	3	17					
ss715608522	ss715608522	Gm10	2	16					
ss715609333	ss715609333	Gm11	3	14					
ss715613704	ss715613704	Gm12	8	17					
ss715612715	ss715612715	Gm12	7	23					
ss715614263	ss715614263	Gm13	3	19	2E-06	X	Glyma.13 g149000	Late embryogenesis abundant (LEA)	SW
ss715624623	ss715624628	Gm16	4	18					
ss715635103	ss715635103	Gm19	1	24	9E-05		Glyma.19 g151900	Hpt domain	SW
ss715635205	ss715635205	Gm19	6	20	0.0004	X	Glyma.19 g163900	AP2 domain protein	SW
Seed Yield per Plant (SYP)									
ss715586641	ss715586641	Gm03	7	30					
ss715602684	ss715602688	Gm08	4	29					
ss715603479	ss715603479	Gm09	0	8					
ss715603626	ss715603626	Gm09	5	27	0.0004	X	Glyma.09 g040000	Response regulator receiver	SY, SW
ss715615670	ss715615670	Gm13	16	35					
ss715618757	ss715618757	Gm14	1	22					
ss715620375	ss715620383	Gm15	6	28	0.0003		Glyma.15 g145200	Response regulator	SY, SW
ss715634241	ss715634241	Gm19	0	14					
ss715637551	ss715637574	Gm20	13	37	0.0002		Glyma.20 g112200	Inner centromere protein	SY, SW

**Fig. 2:**
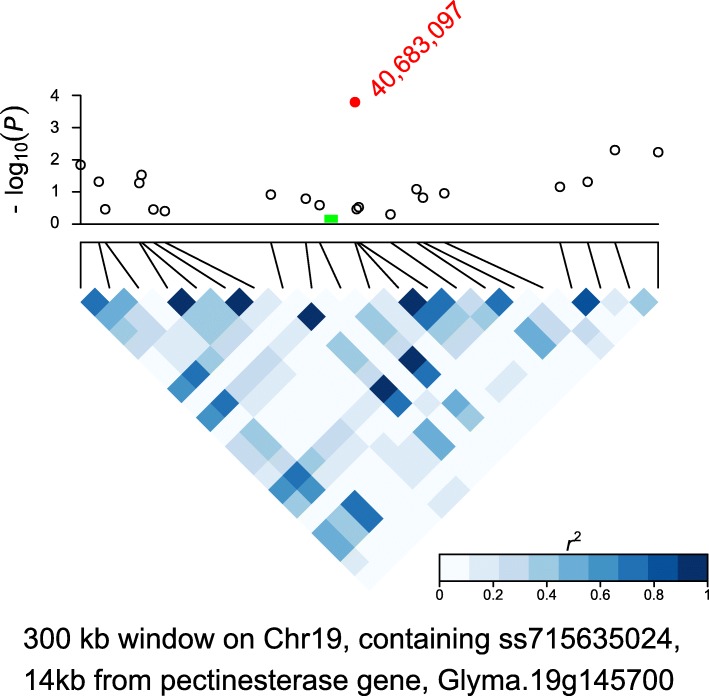
An association region for internode length (IN), on chromosome 19. Top panel: -log_10_ of *P* transformed values from GWAS for IN, within a 300 kb window; bottom panel: LD, measured in r^2^. The most significant SNP is ss715635024 (red dot), at a genomic position of 40,683,097. A candidate gene in this region is Glyma.19 g145700, a pectinestrase, at 14 kb from the significant SNP (location marked in green)

**Fig. 3:**
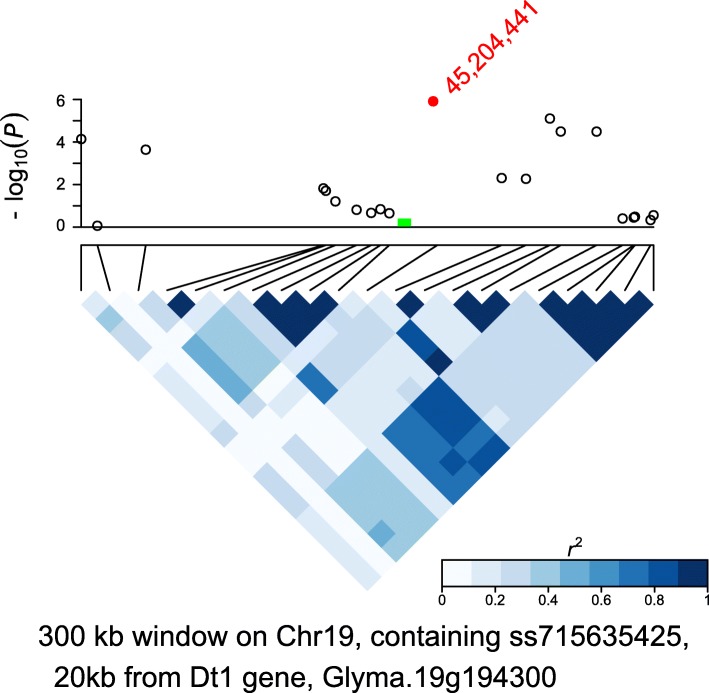
An association region for plant height (PH), on chromosome 19. Top panel: -log_10_ of *P* transformed values from GWAS for IN, within a 300 kb window; bottom panel: LD, measured in *r*^2^. The most significant SNP is ss715635425 (red dot), at a genomic position of 45,204,441. A candidate gene in this region is Glyma.19 g194300, the determinacy (*Dt1*) gene, at 20 kb from the significant SNP (location marked in green)

We evaluated genes around 15 loci associated with IN (Table 2 and Additional files 3 and 4). We note several genes with functional annotations related to shoot related development, including shoot apical meristem (SAM) development and processing of pectin-containing cell walls. A candidate gene Glyma.01 g022500 on chromosome 1 encodes an AP2 domain, which has been shown in other species to be involved in SAM identity [21]. A second candidate gene, Glyma.01 g074000, at a different GWA locus on chromosome 1, encodes a ubiquitin protein ligase; it is homologous to AT2G44950, which encodes a gene involved in meristematic transition from vegetative to reproductive phase (Table 2, Additional files 3 and 4) [22, 23]. On chromosome 16, Glyma.16 g016400, encodes a “No Apical Meristem” (NAM) protein and is homologous to AT3G49530, which is involved in formation of the SAM [24–26]. Similarly, Glyma.19 g002900, on chromosome 19, also encodes a NAM protein. For the second locus on chromosome 19, a candidate gene in that region, Glyma.19 g145700, encodes a pectinesterase (pectin lyase-like protein) (Fig. 2). Another GWA locus on chromosome 19 is near (82 kb) the *Dt1* gene, which is involved in control of flowering time and development of the inflorescence meristem (Fig. 3) [10, 27, 28].

For PH, we note 4 of 10 GWA loci having with functional annotations clearly related to plant growth and development, growth regulation, and regulation of flowering time and development of inflorescence meristem (Table 2 and Additional files 3 and 4). A candidate gene, Glyma.02G245600, encoding Gibberellin-regulated family protein with homologs related to plant height, is located within 10 kb of a significant SNP on chromosome 2 (Additional files 3 and 4). In another association region on chromosome 19, we note that a candidate gene Glyma.19G192400 is homologous to AT2G36450.1, which encodes an ethylene-responsive transcription factor that is involved (in *Arabidopsis*) in plant growth regulation (Additional files 3 and 4) [29, 30] . PH was also associated with the *Dt1* gene on chromosome 19 (overlapping an association region for IN, above) (Fig. 3).

For SW, we note 5 of 14 GWA loci having functional annotations clearly related to seed maturation and seed storage proteins (Table 2 and Additional files 3 and 4). The candidate gene Glyma.02 g046600 on chromosome 2 encodes a histidine kinase protein and is homologous to AT2G17820, which has been shown to be associated with seed maturation during water stress [31]. The candidate gene Glyma.19 g151900 on chromosome 19 encodes a histidine-containing phosphotransfer (Hpt) domain protein and is homologous to AT1G03430.1, which has been reported to play a role in seed size [6]. The second locus is associated with a SNP in high LD (r^2^ > 0.7) on chromosome 19. The candidate gene in that region, Glyma.19 g163900, encodes an AP2-domain protein, which is involved in maintaining seed size, embryo size, seed weight and seed yield [32, 33].

For SYP, we note 3 of 9 GWA loci having functional annotations related to the development maturation, and size of seeds (Table 2 and Additional files 3 and 4). A candidate gene on chromosome 9, Glyma.09 g040000, encode a response regulator receiver. The is related to regulation of seed growth and seed size development [34, 35]. On chromosome 15, the candidate gene Glyma.15 g145200 encodes a response regulator receiver domain, which is related to regulation of seed growth in *Arabidopsis* [36, 37].

### Genome wide epistatic interaction

Tests for SNP-SNP interactions were performed using a linear regression method implemented with PLINK v1.07 [38]. Epistatic tests identified 11, 10, 9, and 3 SNP-SNP interactions associated with IN, PH, SW, and SYP, respectively (Additional file 6). Four SNPs (ss715578706, ss715588448, ss715592908, and ss715593060) on chromosomes 1, 4 and 6 exhibited significant interactions with nine SNPs on 8 other chromosomes for the IN trait (Additional file 6). The main-effect SNP ss715578706 had an additional significant epistatic interaction. For PH, four main-effect SNPs (ss715582993, ss715635454, ss715635458, and ss715635406), on chromosomes 2 and 19, showed significant epistatic interactions with nine SNPs on other chromosomes. For SW, nine significant SNPs (ss715582341, ss715592842, ss715593095, ss715593417, ss715593870, ss715613107, ss715611786, ss715616880, and ss715614051), on chromosomes 2, 6, 12, and 13 interacted with six SNPs on other chromosomes. A main-effect SNP ss715582341 had additional epistatic interactions (Additional file 6). For SYP, three SNPs (ss715603598, ss715603776, and ss715620383) on chromosome 9 and 15 exhibited significant interactions with three SNPs on other chromosomes. In this study, a total of 9, 10, 6, and 3 candidate genes were predicted for IN, PH, SW and SYP, respectively for loci involved in SNP-SNP interactions (Additional file 6). We hypothesize that more SNP-SNP interactions were seen for IN than for SYP because there are likely numerous contributing loci for the complex SYP trait, generally with small effect, and many of these are likely not picked up from our study – and therefore not available for discovery of epistatic effects.

## Discussion

Many QTL mapping studies have provided useful information about approximate genetic locations underlying the genetic control of important agronomic traits in soybean, but these results often have limited mapping resolution. In the current study, we used GWAS to refine the regions previously reported with QTL associations for internode number, plant height, seed weight and seed yield per plant. A collection of 419 diverse soybean PI lines, obtained from the U.S. National Plant Germplasm System, was used to evaluate internode number (IN), plant height (PH), 100 seed weight (SW) and seed yield per plant (SYP). We observed significant genetic differences among genotypes for all four traits, a result connected to the general stability of these traits for each genotype across replicates and locations. We found moderate to high heritability (H^2^) estimates for the traits measured.

GWAS identified 15 and 10 loci associated with IN and PH, respectively (Table 2 and Additional files 3 and 4). These loci were distributed on 11 chromosomes and some of regions overlapped previously reported QTLs for soybean node number and plant height [3, 8, 39–41]. One locus at chromosome 19 was shared by both IN and PH, suggesting these traits are controlled by genes that have pleotropic effects. Indeed, we found a significant phenotypic correlation (0.71) between IN and PH, further indicating the two traits are likely to be at least partly under common control [3]. In our study, both IN and PH were associated with the locus identified by SNP ss715635454, which is in LD with the *Dt1* gene, that has been shown to control flowering and plant height; these traits are often highly correlated [8]. Previous studies have showed the *Dt1* gene to regulate many agronomic traits including plant height and flowering in soybean [8, 42–44]. In soybean, genotypes exhibit either determinate or indeterminate stem growth [45]. The determinate growth habit genotype (which is partly controlled by the recessive *dt1/dt1 alleles*), is produced if the shoot apical meristem (SAM) switches from vegetative to reproductive growth [45]. The presence of the dominant alleles (*Dt1/Dt1*) suppresses the transition to a reproductive inflorescence [45]. Hence, soybean plant height and node production are highly affected by stem growth habit, which in turn has consequences for flowering. Our results provide further evidence in support of the effects of the *Dt1* gene on important agronomic traits like plant height and internode number. Another two loci on chromosome 19 that are associated with IN and PH include candidate genes Glyma.19 g145700 and Glyma.19G187800. Both genes are annotated as having pectinesterase homology and may therefore be involved in processing of cellulose and pectin-containing cell walls [46]. Cellulose and pectin are polysaccharides and are important for plant growth and development, with involvement in cell wall expansion and stem elongation [47]. This is evidenced, for example, by overexpression of a *petunia inflate* pectinesterase in potato plants, which was involved in stem elongation [48]. Pectinesterase has been shown to control cell growth and normal cell elongation in *Arabidopsis* hypocotyls by affecting the degree of pectin methyl-esterification [49].

Through numerous studies, more than 200 QTLs have been identified for SW in soybean (https://soybase.org/), as well as for related traits such as seed size, seed length, seed height and width. In the current study, 14 loci associated with SW were identified. Each locus could explain <3.5% of the phenotypic variation – as might be expected for a complex quantitatively inherited trait. Of the 14 loci identified, seven of them were previously reported as QTL at least once (Additional file 3). The locus on chromosome 2 (ss715582341) showed strong association with SW and seed size-related traits, including seed height and seed width (Additional file 3). This region overlaps QTL for similar traits [50, 51]. Several candidate genes identified on chromosome 19 may have relevance for SW. Glyma.19 g151900 is annotated as a member of the AHP (*Arabidopsis* histidine phosphotransfer) protein family (Additional file 3). AHPs mediate the intracellular response to cytokinins via a two-component phosphorelay signaling pathway from the AHK (*Arabidopsis* histidine kinase) cytokinin receptors to the ARRs (*Arabidopsis* response regulators) and CRFs (cytokinin response factors) [50]. CRFs consist of a subgroup of AP2 transcriptional factors [51]. The sizes of triple cytokinin receptor (AHK2, AHK3, and CRE1/AHK4) loss-of-function mutant seeds were more than doubled due to increased size of the embryo [52]. Similarly, the ahp1,2,3,4,5 “penta” histidine phosphotransfer protein mutation in *Arabidopsis* resulted in larger seeds and embryos [50], suggesting that the homologous AHP Glyma.19 g151900 could also be involved in a cytokinin-mediated seed weight regulating pathway in soybean [6]. The candidate gene Glyma.19 g163900 encodes an AP2 domain protein, analogues of which play significant roles in maintaining seed size, embryo size, seed weight, and seed yield. APETALA2 is involved in regulating in the maternal endosperm in *Arabidopsis* [33] and affects various vegetative organs as well. Further studies will be needed to test the functions and effects of these genes in soybean.

Nine significant loci were identified for seed yield per plant (SYP). The SYP loci on chromosome 9 (ss715603626) and chromosome 15 (ss715620383) were identified within regions overlapping four previously reported QTLs for seed yield [53–55] and four others for seed weight [2, 4, 44, 56] . In addition, several QTLs identified by previous studies tested in different environments were detected in this study, indicating that these seed yield associations are likely stable and might be used to improve soybean seed yield.

For agronomic traits such as IN, PH, SW, and SYP, estimating epistatic loci could help to clarify complex genetic effects of GWAS loci and elucidate other types of interactions such as genotype x environment effects. For internode number, an epistatic candidate gene Glyma.03 g259800 is annotated as a being in the ROP (Rho of Plants)-activated gene family. It potentially interacts with the main gene Glyma.01 g022500 that manifests an AP2 homology (suggesting regulation of SAM identity). ROP-activated genes are reported to play a role in proper cell wall organization during the growth and development of plants [57]. In *A. thaliana*, AP2 is actively involved in vegetative development by regulating the RAP2 (“Related to AP2”) gene expression in stems as well as functioning in reproductive development [58]. Another epistatic candidate gene, Glyma.17 g110300, annotated as an Acetylglucosaminyltransferase protein, interacts with the main-effect gene, Glyma.06 g152000 (a pectinesterase). Furthermore, Glyma.17 g110300 is also homologous to the AT5G33290 gene annotated as a glycosyltransferase superfamily protein shown to play a role in pectin biosynthesis [59]. Pectin is a complex polysaccharide in plant cell wall and it is important for plant growth, development and defense [60]. For plant height, Glyma.02G245600, encodes a gibberellin-regulated family protein, with homologs known to have key function in plant development, while Glyma.19 g194300 encodes a phosphatidylethanolamine binding protein, and has been shown to play key role in regulating of flowering time in soybean and other plants [4, 8, 44]. For seed weight and seed yield significant related main and epistatic genes were identified (Additional file 6). In this GWAS study, an overlap between the locations of main effect and epistatic PH and IN genes suggests that GWES was an effective methodology.

## Conclusions

This study provides a large number of interesting additive and epistatic loci possibly controlling important agronomic traits such as IN, PH, SW, and SYP in soybean. Markers reported here may help in future genomic studies and cultivar development programs.

## Methods

### Plant materials

This study included 419 diverse soybean PI accessions originating from 26 countries, including China, Japan, South Korea and the USA. These genotypes were obtained from the USDA National Plant Germplasm System (http://www.ars-grin.gov; Additional file 5). They were selected for breadth of phenotypic diversity and were derived from three maturity groups (MG): MG I, II, and III.

### Field phenotyping

Soybeans were planted at three locations in central Iowa in 2016 and 2017: the Bruner Research Farm, Agronomy and Agricultural Engineering Research Farm (AAERF), and Burkey Research Farm. The soil type at Bruner Research Farm is Nicollet (poorly drained black loam), AAERF is Webster (silty clay loam) and the Burkey Research Farm is Clarion (well-drained black clay). (Note that in 2016, only PH was measured, as the primary objective in that year was for initial assessment of maturity and disease characteristics.) No fertilizer was applied to experimental plots. At all three sites, in both years, the soybean lines were planted using randomized complete block design with two replications. Each accession was planted in single row plot 0.5 m long, with 40 cm between plots. At full pod (R4) and beginning seed (R5), internode number (IN) per plant were recorded. The internode number of the main stem was taken as the average of three such measurements. Plant height was measured in 2016 and 2017 at physiological maturity from three randomly selected plants per plot, with each measured for main stem length from the soil surface to the tip of the plant. SW and SYP were harvested from one replication at each location. Hundred seed weight was measured for each genotype on a plot basis. SYP was estimated as an average of six plants per plot and seed weight was adjusted to 12% moisture content.

### Genotyping and quality control

The “SoySNP50k” SNP dataset for the panel was described by Song et al. [61]. The SoySNP50k variant data were accessed from SoyBase (https://www.soybase.org/snps/) in spring 2018. The SNP dataset contains 42,506 high confidence SNPs. Of these, 59 SNPs that were unanchored to the Williams 82 v2 reference genome sequence were excluded from further analysis. We used BEAGLE version 3.3.1 with the default parameter settings to impute missing data [62, 63]. Markers with more than 10% missing values were excluded from further analyses. Following imputation, SNPs with a minor allele frequency < 5% were also excluded, yielding 36,140 SNPs for use in GWAS and GWES.

### Marker distribution and linkage disequilibrium

Genome-wide inter-marker distances and chromosome-wide densities were calculated using chromosomal physical lengths, which were obtained from the *Glycine max* Wm82.a2 reference genome. The linkage disequilibrium (LD) was determined as the squared allelic frequency correlation coefficient (r^2^) in the R package synbreed [64]. The r^2^ was computed separately for euchromatic and heterochromatic regions due to significant differences in recombination rate for these two regions. The approximate euchromatic/heterochromatic boundaries for each chromosome were as reported in the SoyBase Wm82.a2 genome browser (https://www.soybase.org). The average LD decay graph was plotted using an R script, which was computed according to Remington et al. [65] r^2^ was calculated only for SNPs with a pairwise distance less than 10 Mbp in either euchromatic or heterochromatic regions of each chromosome. The LD decay rate was determined as the chromosomal distance at the point where the mean r^2^ dropped to half of its maximum value [66].

### Genome-wide association (GWA) and genome wide epistatic (GWE) interaction analysis

The best linear unbiased prediction (BLUP) values for individual accessions were determined using the R package lme4 [67]. This is used to reduce the effects of environmental variation for the measured phenotypic traits. The GWAS analyses were implemented for each trait using GAPIT software [20, 68]. The mixed linear model (MLM) didn’t include population structure as a covariant, as indicated by the Bayesian information criteria (BIC) test of model fit (Additional file 7, [69]). The empirical significance level at *p* < 0.001, determined by 1000 permutations, was used as the significance threshold of SNPs-trait associations as described in Zhang et al. [18]. The genome-wide epistatic interactions between a pair of SNPs were analyzed using the software PLINK version 1.07 [18, 38]. To adjust the multiple comparisons of SNPs, we selected a Bonferroni threshold of α = 0.05.

### Candidate gene prediction

Two sets of gene models were used for identification of candidate genes: the JGI-produced Glyma.Wm82.a2.v1 annotations and the NCBI RefSeq gene models, both called in the GlymaWm82.a2 assembly. Candidate genes in proximity to GWA markers were identified using two approaches: (1) genes within the region circumscribed by the two closest non-significant SNPs on either side of a significant SNP and (2) genes found in the genomic region within 100 kb upstream and downstream of the SNPs with highest association for the trait. All significant SNPs in close proximity were grouped at LD r^2^ > 0.7 [8] to designate candidate regions for quantitative trait loci (QTL), and only the peak SNPs within each LD block were maintained.

### Statistical analysis

The model for phenotypic data collected for each trait was *Y*_ijk_ = *μ* + *g*_i_ + *l*_*j*_ + *(gl)*_ij_ + *b*_*k(*j)_ + *e*_ijk_, where μ is the overall population mean, g_i_ is the genetic effect of the i^th^ genotype, *l*_*j*_ is the effect of j^th^ location, *(gl)*_*ij*_ is the interaction effects between the *i*^*th*^ genotype and *j*^*th*^ location, b_k(j)_ is the effect of the block within the j^th^ location, and e_ijk_ is the residual effect including random error following *N* (0, *σ*^*2*^_*e*_). Broad sense heritability for each trait was estimated on an entry mean basis using the equation, H^2^ = *σ* ^2^_g_ */* [*σ* ^2^_g_ *+* (σ ^2^_g*l_/l) + (σ ^2^_e_*/*rl)], where *σ* ^*2*^_*g =*_ genotypic variance, σ ^2^_g*l_ = genotype by location interaction variance**,** l = number of locations, and r = number of replicates, and σ^2^_e_ is the error variance assuming all effects are random. We computed variance components estimation was computed using SAS version 9.3 (SAS Institute Inc., Cary, NC).

## Additional files

Additional file 1: **Figure S1.** Frequency distribution of observation of internode number, plant height, seed weight, and seed yield per plant in soybean. (DOCX 79 kb)

Additional file 2: **Figure S2.** The mean level of linkage disequilibrium (LD) decay rate in euchromatic and heterochromatic chromosome regions. The mean decay of LD was estimated as squared correlation coefficient (r2) using all pairs of loci within 10 Mb of physical distance. The x-axis shows the distance between markers pairs in Mb and the y-axis shows LD in r2. The red line denotes euchromatic region and black line denotes the heterochromatic region. The dashed grey line shows the position where r2 dropped to half of its maximum value. (DOCX 53 kb)

Additional file 3: Summary of GWAS regions, genes of interest, and overlapping QTLs. Extends Table 2, with SNP position data, *P*-values, distances to SNPs, and additional information about functional annotations. Counts of candidate genes are given for regions defined by two methods: by flanking non-significant markers around significant SNP(s), and within a window defined by the significant SNP(s) plus 100 kb before and after the significant SNP(s). (XLSX 22 kb)

Additional file 4: Genes within GWAS regions along with gene annotations. Genes are given for regions defined by two methods: by flanking non-significant markers around significant SNP(s), and within a window defined by the significant SNP(s) plus 100 kb before and after the significant SNP(s). (XLSX 150 kb)

Additional file 5: PI lines used in the study, arranged based on maturity group and country of origin. (XLSX 20 kb)

Additional file 6: Significantly associated genome wide epistatic (SNP-SNP) interactions and associated candidate genes. (XLSX 14 kb)

Additional file 7: Bayesian Information Criterion (BIC) mixed linear model with principal components (PCs) applied for association analysis of internode, plant height, seed weight and seed yield per plant. (DOCX 14 kb)

## Data Availability

All data associated with this study are available under the “Additional files” data sets. All accessions used in this study are indicated in Additional file 5, with “Plant Introduction” numbers referencing germplasm available through the U.S. National Plant Germplasm System: https://npgsweb.ars-grin.gov.

## Abbreviations

BLUP: best linear unbiased prediction
GWA: genome-wide association
GWAS: genome-wide association study
GWE: genome-wide epistatis
GWES: genome-wide epistatic study
IN: internode number
LD: linkage disequilibrium
MLM: mixed linear model
NAM: no apical meristem
PH: plant height
PI: plant introduction
QTL: qualitative trait locus
SAM: shoot apical meristem
SNP: single nucleotide polymorphismm
SW: seed weight
SYP: seed yield per plant
