# Supplementary material for: Genome-wide associations and epistatic interactions for internode number, plant height, seed weight and seed yield in soybean
Source: BMC Genomics. 2019 Jun 26;20:527. doi: 10.1186/s12864-019-5907-7 (PMC6595607; doi:10.1186/s12864-019-5907-7)
Supplement: Supplementary file 1 — Figure S1. Frequency distribution of observation of internode number, plant height, seed weight, and seed yield per plant in soybean. (DOCX 79 kb) [file 12864_2019_5907_MOESM1_ESM.docx]

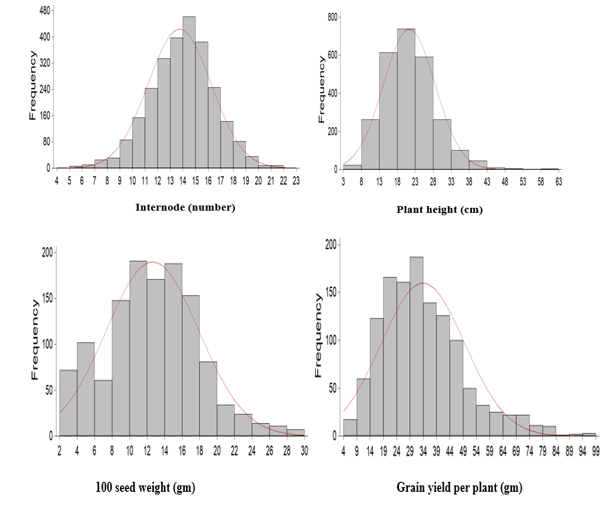


**Additional figure1: Frequency distribution of observation of internode number, plant height, seed weight, and seed yield per plant in soybean.**
