# Supplementary material for: Genome-wide associations and epistatic interactions for internode number, plant height, seed weight and seed yield in soybean
Source: BMC Genomics. 2019 Jun 26;20:527. doi: 10.1186/s12864-019-5907-7 (PMC6595607; doi:10.1186/s12864-019-5907-7)
Supplement: Supplementary file 2 — Figure S2. The mean level of linkage disequilibrium (LD) decay rate in euchromatic and heterochromatic chromosome regions. The mean decay of LD was estimated as squared correlation coefficient (r2) using all pairs of loci within 10 Mb of physical distance. The x-axis shows the distance between markers pairs in Mb and the y-axis shows LD in r2. The red line denotes euchromatic region and black line denotes the heterochromatic region. The dashed grey line shows the position where r2 dropped to half of its maximum value. (DOCX 53 kb) [file 12864_2019_5907_MOESM2_ESM.docx]

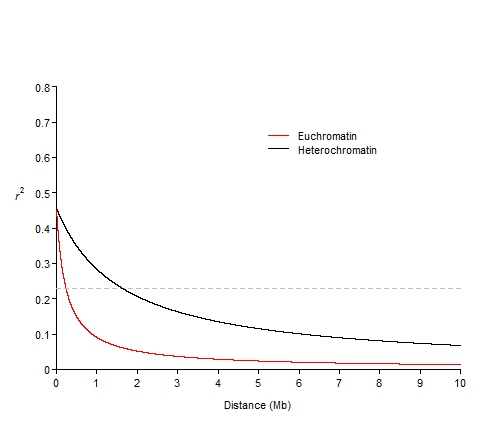


Additional Figure 2. The mean level of linkage disequilibrium (LD) decay rate in euchromatic and heterochromatic chromosome regions. The mean decay of LD was estimated as squared correlation coefficient (r^2^) using all pairs of loci within 10 Mb of physical distance. The x-axis shows the distance between markers pairs in Mb and the y-axis shows LD in r^2^. The red line denotes euchromatic region and black line denotes the heterochromatic region. The dashed grey line shows the position where r^2^ dropped to half of its maximum value.
