# Supplementary material for: Genome-wide associations and epistatic interactions for internode number, plant height, seed weight and seed yield in soybean
Source: BMC Genomics. 2019 Jun 26;20:527. doi: 10.1186/s12864-019-5907-7 (PMC6595607; doi:10.1186/s12864-019-5907-7)
Supplement: Supplementary file 7 — Bayesian Information Criterion (BIC) mixed linear model with principal components (PCs) applied for association analysis of internode, plant height, seed weight and seed yield per plant. (DOCX 14 kb) [file 12864_2019_5907_MOESM7_ESM.docx]

**Bayesian Information Criterion (BIC*) mixed linear model with principal components (PCs) applied for association analysis of Internode, plant height, seed weight (SW) and seed yield per plant (SYP).**

| No. of PCs | Internode | Plant height | SW | SYP |
| --- | --- | --- | --- | --- |
| 0 | -746.12 | -1172.61 | -1066.08 | -1560.58 |
| 1 | -748.83 | -1175.44 | -1063.08 | -1562.57 |
| 2 | -751.66 | -1177.03 | -1065.43 | -1560.95 |
| 3 | -754.72 | -1179.71 | -1068.45 | -1563.97 |
| 4 | -756.87 | -1180.79 | -1071.44 | -1566.41 |
| 5 | -757.89 | -1183.51 | -1073.23 | -1568.18 |
| 6 | -760.53 | -1186.38 | -1074.90 | -1570.71 |
| 7 | -763.60 | -1188.83 | -1077.88 | -1573.65 |
| 8 | -766.60 | -1191.87 | -1080.55 | -1576.72 |
| 9 | -769.68 | -1194.76 | -1083.58 | -1579.76 |
| 10 | -772.27 | -1196.94 | -1085.04 | -1582.35 |

*BIC: larger is more significant
